# The *daf-2* insulin receptor functions in *C. elegans* embryo elongation

**DOI:** 10.17912/micropub.biology.000117

**Published:** 2020-01-13

**Authors:** Annu Suresh, Bruce Wightman

**Affiliations:** 1 Biology Department, Muhlenberg College, 2400 Chew St., Allentown, PA 18104 USA

**Figure 1:**
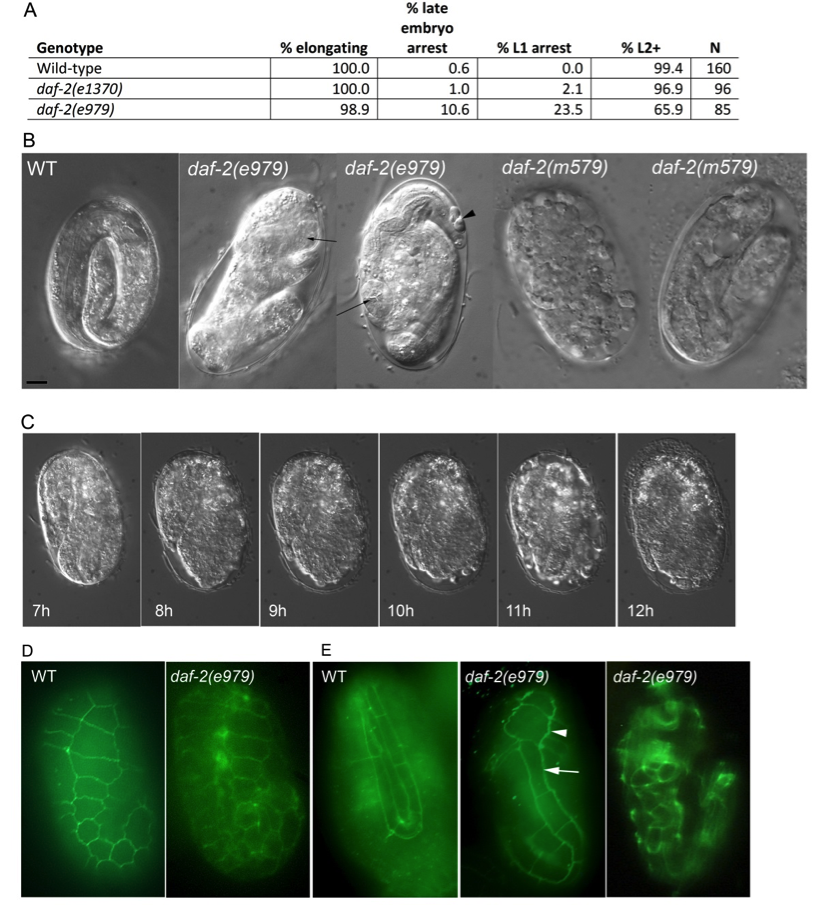
Arrest of *daf-2(e979)* embryos. A.Embryos grown at 25^o^C showing the percentage that initiate elongation, arrest due to embryonic lethality after elongation, arrest at or after hatching as fully-elongated animals, and proceed on to L2 and later stages. Wild-type animals all proceed to adult, but both *daf-2(e1370)* and *daf-2(e979)* animals that proceed past the L1 stage arrest as dauer larvae. N is number of embryos scored. B. DIC micrographs of post-elongation wild-type, *daf-2(e979),* and *daf-2(m579)* embryos. Second panel: arrow identifies head portions that have failed to elongate. Third panel: arrow identifies a grossly normal pharynx; arrowhead identifies cellular debris outside the body of the embryo. C. Still DIC micrographs from time-lapse of a single *daf-2(e979)* undergoing elongation failure and embryonic rupture over a 5 hour period at 25^o^C. D. Wild-type (WT) and *daf-2(e979)* fixed embryos prior to elongation showing the outlines of epithelial (hypodermal) cells using MH27 immunofluorescence and apparently normal patterning. E. MH27 immunofluorescence of elongating embryos. Note the partially elongated H2 lateral seam cell (arrow) and unelongated anterior H1 seam cell (arrowhead) in middle image. Retracted hypodermal seam cells are evident in the later embryo that has failed to elongate shown in the image at right. Scale bar = 5uM.

## Description

The *daf-2* gene codes for the singular insulin-like growth factor receptor in *C. elegans*, acting as a control point in multiple developmental and physiological pathways that depend on the integrated response to at least forty different insulin-like peptides (Murphy and Hu, 2013). The functions of *daf-2* in dauer formation, longevity, germ-line, and early larval arrest have been extensively studied. A strong *daf-2* allele, *e979,* is homozygous viable at 15^o^C, despite a partially penetrant embryonic lethal phenotype at 25^o^C (Gems et al., 1998). The presumed null allele, *m65*, does not produce fertile homozygous adults due to 100% dauer arrest, and does not display embryonic lethality due to maternal rescue from heterozygous mothers. While several *daf-2* alleles display low penetrance embryonic lethality at 25^o^C, the *e979* allele displays the most penetrant embryonic arrest phenotype (Gems et al., 1998; Patel et al., 2008), making it the best choice to investigate the embryonic requirement for insulin signaling. The *e979* mutation causes a C146Y substitution that is thought to interrupt an existing disulfide bond interaction with C181, destabilizing the typical fold of the insulin receptor, and impairing the L1 domain’s function in ligand binding (Patel et al., 2008).

To investigate the role of insulin-like signaling in embryonic development, we examined *daf-2(e979)* and parallel N2 wild-type and *daf-2(e1370)* controls at 25^o^C. The canonical *e1370* allele causes a strong dauer arrest phenotype, but a very low penetrance embryonic arrest phenotype. While 98.9% of *daf-2(e979)* embryos reached early elongation midway through embryogenesis (comma stage), 10.6% of embryos arrested without hatching (Fig. 1A). Similar to previous reports, 23.5% of *daf-2(e979)* animals arrested development as fully-elongated L1 animals at or after hatching (Gems et al., 1998; Baugh, 2013). Examination of arrested *daf-2(e979)* embryos by DIC microscopy revealed that they had some of the features of late embryogenesis, such as an apparently normal pharynx, but had failed to elongate properly (Fig. 1B). We followed five control wild-type embryos and six *daf-2(e979)* embryos by time-lapse microscopy over an 8 hour period at 25^o^C beginning at the mid-embryogenesis comma stage. While all of the wild-type and five of the *daf-2(e979)* embryos exhibited normal elongation and morphogenesis, one *daf-2(e979)* embryo failed to elongate, beginning just before the two-fold stage (Fig. 1C). The failing embryo twitched normally at this stage, indicating that it had functional muscles, but was unable to elongate past the two-fold length and retracted somewhat over a one hour period. After several hours, blebs appeared at the anterior end of the embryo and it eventually ruptured. These observations suggest that insulin-like signaling plays a role in embryonic elongation in *C. elegans*. A role for *daf-2* in embryo elongation was previously suggested based on synthetic genetic interactions of other *daf-2* alleles with the *let-502* Rho-binding kinase (Piekny, et al., 2000), but has not been described for *daf-2* mutations alone.

The embryonic elongation process is driven by the migration, fusion, and contraction of the hypodermal epithelium (Priess and Hirsh, 1986; Costa et al., 1998; Chisholm and Hardin, 2005). Circumferential actin microfilaments connect the longitudinal margins of the belt desmosomes surrounding each hypodermal cell and contractile activity leads to the change in hypodermal cell shape seen in elongation. Therefore, elongation depends on both the mechanistic contraction of actin microfilaments and the arrangement and differentiation of the hypodermal cells themselves.

We investigated the patterning of hypodermal cells of N2 wild type and *daf-2(e979)* embryos grown at 25^o^C prior to and during embryonic elongation using indirect immunofluorescence. The adherens junctions of the hypodermal cells were stained using an MH27 primary monoclonal antibody, which outlines the perimeters of the cells (Fig. 1DE). We examined 31 N2 wild-type and 59 *daf-2(e979)* embryos grown at 25^o^C at comma stage prior to elongation (Fig. 1D). All *daf-2(e979)* embryos examined showed hypodermal patterning that was similar to wild-type. Ventral views (not shown) revealed no difficulties with ventral enclosure and dorsal fusion of hypodermal cells appeared normal. We were not able to determine for certain whether anterior enclosure was normal in all embryos. Most embryos (58/59) that were undergoing elongation displayed a normal pattern of hypodermal cells. One *daf-2(e979)* embryo appeared to have elongated lateral seam cells over much of the body, but the H0 and H1 seam cells of the head were not elongated (Fig. 1E), suggesting that this embryo may have been in the process of failing to elongate. Later embryos that were failing to elongate showed hypodermal patterning anomalies including retracted seam cells (Fig. 1E), but it was unclear whether this was a cause or an effect of elongation failure. Taken together, these results suggest that *daf-2* may play a role in the mechanistic process of elongation, but not in the initial differentiation or patterning of the hypodermal cells themselves.

We considered the possibility that the elongation defect phenotype associated with *e979* could reflect a strain-specific effect due to another mutation in the DR 1942 background, despite three rounds of back-crossing. Embryonic lethality was temperature-sensitive (0/146 embryos arrested at 15^o^C; 0.5% reported in Gems et al., 1998), as one would expect for a defect associated with a loss of insulin-signaling. We also examined hundreds of embryos grown at 25^o^C in weaker *daf-2(m41)* and *daf-2(m579)* mutants to look for rare arrested embryos with phenotypes similar to *daf-2(e979)* embryos. The *m41* and *m579* mutations cause 2.7% and 4.4% embryonic arrest phenotypes, respectively (Gems et al., 1998). For *m579* mutant embryos, 1/9 arrested as a morphologically-normal 4-fold embryo, 4/9 arrested as post-elongation embryos with extensive necrosis (Fig. 1B, rightmost panel), 2/9 arrested as two or three-fold embryos with apparent elongation failures (Fig. 1B, second from right panel), and 2/9 arrested as amorphous embryos for which elongation could not be judged. For *m41* embryos, 10/59 arrested as post-elongation embryos with extensive necrosis, 4/59 arrested as two or three-fold embryos with apparent elongation failures, and 41/59 arrested as amorphous embryos for which elongation could not be judged. Although examination of these two weaker alleles did reveal embryos with apparent elongation defects, neither phenocopied the *e979* embryonic arrest phenotype exactly. Our results are consistent with a role for *daf-2* insulin signaling in the embryonic elongation process, however the phenotypic consequences of *daf-2* loss-of-function appear to vary among different alleles.

## Reagents

Wild-type N2, DR 1942 *daf-2(e979),* CB 1370 *daf-2 (e1370),* DR 1564 *daf-2(m41),* and DR1566 *daf-2(m579)* nematode strains were obtained from the *Caenorhabditis Genetics Center*. The MH27 mouse anti-AJM-1 cell junction protein monoclonal supernatant was obtained from the Developmental Studies Hybridoma Bank (AB_531819). The Alexa Fluor 488 goat anti-mouse secondary antibody was obtained from Thermo Fisher (A-11001). Indirect immunofluorescence was performed by preparing embryos from mixed cultures by bleach treatment, followed by paraformaldehyde fixation and permeabilization (Finney and Ruvkun, 1990). Both primary and secondary antibodies were diluted at 1:500. DIC images were captured from a Nikon Eclipse TE2000U inverted microscope using a 60X objective, Nikon NIS software and a PC computer. Immunofluorescence images were captured from a Nikon Optiphot UD2 microscope using a 40X objective, Nikon NIS software, and a PC computer.
